# *Staphylococcus* Strains in Atopic Dermatitis in Children: Toxins Production and Resistance Properties

**DOI:** 10.3390/life15071120

**Published:** 2025-07-17

**Authors:** Asya Kudryavtseva, Fyodor Fluer, Lusine Khachatryan, Svetlana Makarova, Oksana Osipenko, Elena Ryzhii, Sergei Titarev, Denis Zaslavsky, Katerina Gelezhe

**Affiliations:** 1 Escuela de Medicina y Centro de Estudios e Investigación en Salud y Sociedad (CEISS), Facultad de Ciencias Médicas, Universidad Bernardo O’Higgins, Santiago 8370993, Chile; asya.kudryavtseva@psh.ubo.cl; 2 Molecular Basis of Pathogenicity Laboratory, Bacterial Infection Department, Gamaleya Research Centre of Epidemiology and Microbiology, 123098 Moscow, Russia; 3 Department of Pediatric Diseases, N.F. Filatov Clinical Institute of Children Health, I.M. Sechenov First Moscow State Medical University (Sechenov University), 119270 Moscow, Russia; 4 National Medical Research Center for Children’s Health, 119296 Moscow, Russia; 5 My Doctor Family Medical Center LLC, 623701 Ekaterinburg, Russia; 6 Anatomy and Histology Department, Fukushima Medical University, Fukushima 960-1295, Japan; eryzhii@fmu.ac.jp; 7 Department of Dermatovenereology, Saint-Petersburg State Pediatric Medical University, 194100 St. Petersburg, Russia; 8 Laboratory of Molecular Immunology, Immunology and Allergology Department, I.I. Mechnikov Vaccine and Serum Research Institute, 105064 Moscow, Russia

**Keywords:** atopic dermatitis, *Staphylococcus epidermidis*, *Staphylococcus aureus*, enterotoxins, toxic shock syndrome toxin-1, antibiotic resistance, children

## Abstract

*Staphylococcus* spp. skin colonization is involved in the pathogenesis of atopic dermatitis (AD). While coagulase-positive *Staphylococcus aureus* strains are known to worsen symptoms, the role of coagulase-negative staphylococci (CoNS) remains controversial. Further research is needed to clarify the pathogenicity of CoNS in AD patients. A study involving 329 children with AD (mean age: 4.89 years) assessed the frequency of staphylococcal colonization on affected skin, along with the toxin-producing properties and antibiotic resistance of isolated strains. Mild AD: Predominantly colonized by CoNS (especially *S. epidermidis*). Moderate/Severe AD: Showed a significant increase in *S. aureus* colonization. CoNS (including *S. epidermidis*) could produce enterotoxins (A, B, C) and toxic shock syndrome toxin-1 (TSST-1), though less frequently than *S. aureus* strains. In severe AD, the number of toxin-producing CoNS strains (especially enterotoxin A producers) was higher than in mild AD, and the number of non-toxin-producing strains was lower. CoNS exhibited higher resistance rates than *S. aureus*. Methicillin-resistant *S. epidermidis* (MRSE): 23.4%. Methicillin-resistant *S. aureus* (MRSA): 1.27%. CoNS may contribute to AD pathogenesis through toxin production (exacerbating inflammation) and antibiotic resistance (limiting treatment options). Severe AD may involve a synergistic effect between *S. aureus* and toxin-producing CoNS.

## 1. Introduction

Atopic dermatitis (AD) is a chronic inflammatory skin disorder with a complex, heterogeneous etiology, involving impaired skin barrier function, intradermal and systemic T-lymphocyte activation, and increased susceptibility to cutaneous infections [1]. Typically, skin of AD patients is colonized by *Staphylococcus aureus*, a coagulase-positive staphylococcus (CoPS) [2,3]. *S. aureus* exacerbates AD through expression of virulence factors that trigger disease flares [4], contribution to chronic relapsing course of the disease, and potential resistance to anti-inflammatory corticosteroid treatment [5]. Factors promoting *S. aureus* colonization in AD include Th2/Th17 cytokines overexpression, dysregulation of antimicrobial peptides (e.g., HNP1 and β-defensins), microbial dysbiosis, and skin barrier defects [6]. Bacterial toxins further perpetuate inflammation by altering interleukin secretion, creating a self-sustaining inflammatory cycle [7]. CoNS such as *Staphylococcus epidermidis* also colonize patients with AD, but the role of these bacteria in the disease remains poorly understood [8].

*S. epidermidis* is a key component of the normal skin microbiota. During AD flares, *S. epidermidis* colonization increases compared to other commensals [9,10], suggesting a potential compensatory role in suppressing *S. aureus* overgrowth. *S. epidermidis* can modulate host immune responses and prevent pathogenic microorganism invasion by stimulating keratinocytes to produce endogenous antimicrobial peptides and by independently secreting bacteriocins with a potent antimicrobial activity [11]. In murine AD models, *S. epidermidis*-derived vesicles downregulate pro-inflammatory genes (TNFα, IL1β, IL6, IL8 and iNOS), upregulate human β-defensins 2 and 3, and enhance resistance to *S. aureus* colonization [12]. Additionally, *S. epidermidis* produces specific compounds such as serine proteases and phenol-soluble modulins that inhibit biofilm formation and restrict *S. aureus* colony growth [13]. These observations support the potential protective role of CoNS in AD [14].

Despite its beneficial roles, *S. epidermidis* possesses virulence factors that may exacerbate AD. It has the ability to produce enterotoxins that function as superantigens [15]. Pathogenicity islands, containing genes for staphylococcal enterotoxin B (SEB), have been identified in *S. epidermidis* phages [16]. Staphylococcal superantigens can stimulate Th2 lymphocytes to produce interleukin (IL)-31, which suppress filaggrin expression, increase pro-inflammatory cytokines production, activate basophils, and induce intense pruritus [17,18]. Staphylococcal enterotoxins may also act as allergens [19]. Clinical studies have shown positive correlations between *S. epidermidis* colonization density and serum anti-SEB IgE levels [20]. Nevertheless, no studies have directly confirmed enterotoxin production by CoNS strains isolated from AD patients’ skin. The potential adverse effects of *S. epidermidis* and other CoNS on the course of AD can be aggravated by their multi-drug-resistant properties [21,22]. Our study aims to provide a more detailed investigation of these potentially harmful characteristics of CoNS and compare them with those of *S. aureus* strains in a pediatric cohort of AD patients.

## 2. Materials and Methods

We conducted an observational cross-sectional study at the outpatient department of the University Children’s Clinical Hospital, First Moscow State Medical University. Potential participants were initially identified by pediatricians, with subsequent evaluation by allergists and a dermatologists to confirm AD diagnosis using the Hanifin and Rajka criteria and assess eligibility.

The study included children aged 2 to 18 years with either recently or previously diagnosed AD, regardless of disease duration, severity, or comorbid allergic conditions. Key inclusion criteria required visible AD lesions in both antecubital fossae. Patients were divided into three groups (mild AD, moderate AD, and severe AD) based on their SCORAD (SCORing Atopic Dermatitis) index, a standardized clinical tool used to objectively measure the severity of AD by evaluating three key aspects: extent, intensity, and subjective symptoms [23]. Group definitions were mild AD (SCORAD ≤ 25), moderate AD (25–50), and severe AD (≥50). Exclusion criteria comprised the following: age < 2 years; active skin infection; immunodeficiency disorders, recent (within 1 month) use of immunosuppressants (including oral corticosteroids), systemic/topical antibiotics, topical anti-inflammatory medications, or moisturizers.

Trained allergist/dermatologists obtained bilateral antecubital fossa swabs for *Staphylococcus* spp. identification. Samples were processed using MicroScan WalkAway plus System (Beckman-Coulter, Inc., Brea, CA, USA) for microb ial identification and antibiotic susceptibility testing.

Isolated staphylococcal strains were placed on a liquid nutrient medium—Casman’s salt composition medium with our modifications. The acidic casein hydrolysate (Difco) was replaced with an enzymatic casein hydrolysate from the Gamaleya Research Centre of Epidemiology and Microbiology, Moscow, Russian Federation, and 1% BHI was added. Subsequent cultivation was performed on a rotary shaker at 210 rpm for 24 h at 37 °C. For cultivation, 50 mL tubes were used, into which 4.5 mL of culture medium was added. Bacterial cells were removed by centrifugation 9218× *g* for 15 min (centrifuge Janetzki K-24, fixed-angle rotor 6 × 35 mL) and obtained supernatant was heated for 30 min at 100 °C. Detection of staphylococcal enterotoxin C (SEC) was conducted using a double diffusion method in a gel with monospecific serum to the SEC [24]. Detection of staphylococcal enterotoxin A (SEA), and staphylococcal enterotoxin B (SEB) was determined with an enzyme-linked immunoassay test kit with a sensitivity of 2.0 ng/mL for SEA and 1.0 ng/mL for SEB [25,26]. Toxic shock syndrome toxin 1 (TSST-1) was determined by using an enzyme immunoassay kit with a sensitivity of 10.0 ng/mL [27].

We evaluated colonization patterns (CoNS vs. CoPS), toxin production profiles, and antibiotic resistance rates with comparative analysis across AD severity groups.

## 3. Results

### 3.1. Microbial Isolation Patterns

Our study included 329 children with AD (median age 4.89 years, IQR 2–18). *Staphylococcus* spp. colonization was identified in 244 participants (74.4%), yielding 300 isolated staphylococcal strains:

○ *S. aureus*: 160 strains (53.3%).
○ CoNS: 140 strains (46.6%):
  ▪ *S. epidermidis*: 87 (29.0%);
  ▪ *S. haemolyticus*: 22 (7.3%);
  ▪ *S. hominis*: 15 (5.0%);
  ▪ *S. capitis*: 7 (2.3%);
  ▪ *S. warneri*: 5 (1.7%);
  ▪ *S. cohnii*: 2 (0.7%);
  ▪ Single isolates of *S. simulans* and *S. saprophyticus*.

We detected poly-staphylococcal colonization in 52 patients (15.8%), with the following associations:

○ Most frequent: *S. aureus* + *S. epidermidis* (*n* = 29);
○ Moderate frequency: *S. epidermidis* + *S. haemolyticus* (*n* = 5), *S. aureus + S. haemolyticus* (*n* = 4), *S. epidermidis + S. hominis* (*n* = 4);
○ Rare associations (n = 1 each): *S. aureus* + *S. saprophyticus*/*capitis*, *S. epidermidis* + *S. warneri*/*cohnii*/*capitis*, *S. haemolyticus* + *S. capitis*;
○ Triple colonization: *S. aureus* + *S. epidermidis* + *S. haemolyticus*/*hominis*.

No staphylococci were isolated from 85 AD cases (25.8%).

Mean SCORAD index in patients with *S. aureus* skin colonization was 54.0 ± 4.9, with CoNS skin colonization was 41.4 ± 6.3, and with no staphylococcal growth was 43.2 ± 6.4. In patients with *S. aureus* + CoNS co-colonization, the mean SCORAD index was 51.7 ± 4.6. Statistical comparisons revealed significantly higher SCORAD among patients with *S. aureus* skin colonization compared with CoNS skin colonization or no staphylococcal growth (*p* = 0.0066 and *p* = 0.011).

Among patients with mild AD, CoNS represented the predominant skin colonizers (*p* < 0.05). In moderate AD cases, we observed comparable detection rates of CoNS and *S. aureus* (*p* < 0.05). The colonization pattern shifted markedly in severe AD, where *S. aureus* became the predominant species (*p* < 0.05). Statistical analysis revealed two significant trends in the severe AD subgroup: a progressive decline in CoNS detection alongside a concurrent increase in *S. aureus* colonization (both *p* < 0.05). Patients showing no staphylococcal growth were predominantly diagnosed with mild AD (Table 1).

### 3.2. Toxin-Producing Properties of CoNS vs. CoPS

The toxin-producing properties of 83 staphylococcal strains were investigated, including 32 CoPS (*S. aureus*) and 51 CoNS. The CoNS group comprised 30 *S. epidermidis*, 8 *S. haemolyticus*, 7 *S. hominis*, 3 *S. warneri*, 2 *S. capitis*, and 1 *S. simulans* strain. All strains exhibited the ability to produce at least one toxin, with many strains producing multiple toxins simultaneously.

Among *S. aureus* strains, 78.1% (25/32) produced more than one toxin: 18.75% (6/32) synthesized two toxins, 46.9% (15/32) three, and 12.5% (4/32) expressed all four tested toxins. Similarly, 47.0% (24/51) of CoNS strains produced several toxins: 9.8% (5/51) produced two, 31.4% (16/51) three, and 5.8% (3/51) all four toxins. Non-toxigenic strains were observed in 9.0% (3/32) of *S. aureus* and 15.6% (8/51) of CoNS isolates, with no statistically significant difference (*p* > 0.05).

SEB and TSST-1 were the most frequently detected toxins in staphylococcal filtrates. In undiluted samples, SEB was identified in 87.9% of *S. aureus*, 73.3% of *S. epidermidis*, and 66.7% of other CoNS strains, with no significant difference in detection rates. However, at a 1:10 dilution, SEB was significantly more prevalent in *S. aureus* (63.6%) compared to *S. epidermidis* (16.6%) and other CoNS (23.8%) (*p* < 0.001). These results suggest that, under laboratory conditions, *S. aureus* may produce higher amounts of SEB compared to other staphylococcal strains.

For TSST-1, no significant difference was observed between *S. aureus* (87.9% undiluted, 69.7% at 1:10) and *S. epidermidis* (66.7% undiluted, 53.3% at 1:10), indicating comparable production levels. However, other CoNS strains exhibited significantly lower TSST-1 detection rates (47.61% undiluted, 28.57% at 1:10) compared to *S. aureus* (*p* < 0.01). Neither SEB nor TSST-1 was detectable in the filtrates of staphylococci strains at a 1:50 dilution.

SEA was the second most prevalent toxin. No significant differences were observed in undiluted filtrates among *S. aureus* (78.8%), *S. epidermidis* (73.3%), and other CoNS (76.2%). At a 1:10 dilution, SEA remained highly detectable in *S. aureus* (75.6%) and *S. epidermidis* (70.0%), but its prevalence dropped in other CoNS (47.6%, *p* < 0.05 vs. *S. aureus*). Notably, in *S. aureus* filtrates SEA remained detectable even at 1:50 dilution (24.2%), whereas in *S. epidermidis* filtrates SEA was not detected at this dilution. In a single *S. simulans* strain filtrate (4.7% of other CoNS), SEA was detected at 1:50, suggesting that some CoNS strains can generate SEA in quantities comparable to *S. aureus*.

**Figure 1:**
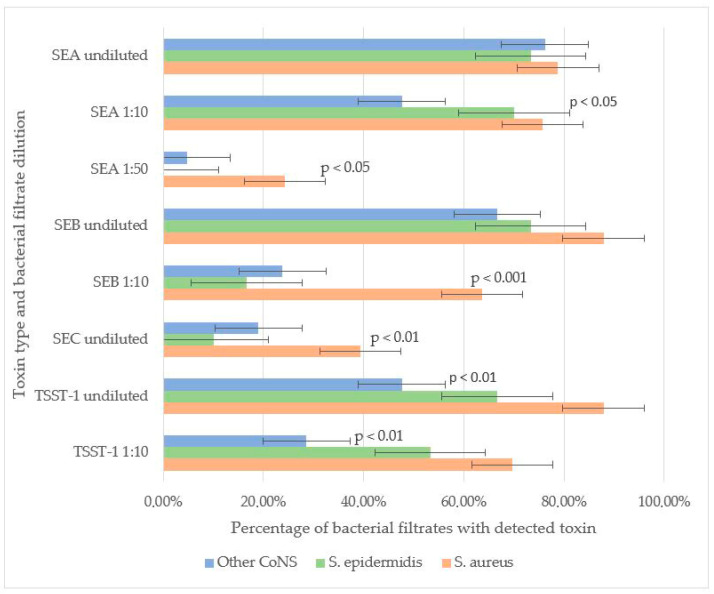
Toxin-producing properties of *Staphylococcus* spp. SEA was detected at similar frequencies in undiluted bacterial filtrates among all staphylococci. In the 1:10 dilution, SEA was detected at similar frequencies in *S. aureus* and *S. epidermidis* strains, and less frequently among other CoNS species (*p* < 0.05). In the 1:50 dilution, SEA was detected more frequently in S. aureus strains compared to CoNS, including *S. epidermidis* (*p* < 0.05). SEB was detected at similar frequencies in undiluted filtrates. In the 1:10 dilution, it was detected more frequently in *S. aureus* strains compared to CoNS, including S. epidermidis (*p* < 0.001). SEC was detected more frequently in *S. aureus* filtrates compared to *S. epidermidis* filtrates (*p* < 0.01). TSST-1 was detected at similar frequencies in undiluted filtrates and in the 1:10 dilution of both S. aureus and *S. epidermidis*. However, other CoNS strains exhibited significantly lower TSST-1 detection rates (in both undiluted and 1:10 filtrates) compared to *S. aureus* (*p* < 0.01).

SEC was the least frequently detected toxin. It was more prevalent in *S. aureus* (39.4%) than in *S. epidermidis* (10.0%, *p* < 0.01) or other CoNS (19.0%). SEC detection in diluted filtrates was not performed (Figure 1).

**Figure 2:**
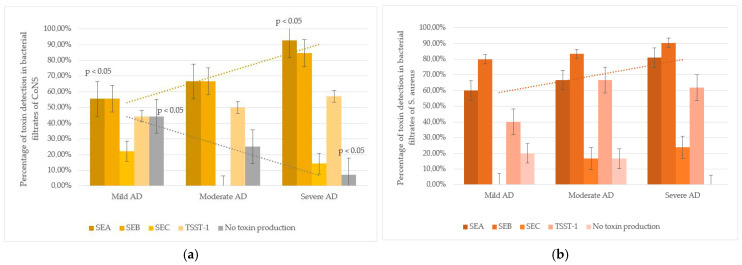
*Staphylococcus* spp. toxins production and AD severity: (**a**) CoNS strains (including *S. epidermidis*): toxin-producing properties were studied in 51 strains (mild AD—13, moderate AD—18, severe AD—20). In mild AD, CoNS produced SEA less often compared to severe AD group (*p* < 0.05). The number of non-toxin-producing strains was significantly higher among mild AD compared to severe AD (*p* < 0.05). (**b**) *S. aureus* strains: toxin-producing properties were studied in 32 strains (mild AD—5, moderate AD—6, severe AD—21). The strains had pronounced toxic properties, regardless of the severity of the disease.

An intriguing trend was observed in CoNS strains: the frequency of SEA detection in culture filtrates was higher in severe AD subgroup compared to mild AD. In mild AD, 53.8% (7/13) of CoNS strains produced SEA; in moderate AD, 61.1% (11/18); and in severe AD, 90.0% (18/20). The difference between mild and severe AD cases was statistically significant (*p* < 0.05). Additionally, non-toxigenic CoNS strains, in whose culture filtrates none of the tested toxins were detected, were more frequent in mild AD (44.4%, 4/9) than in moderate (25.0%, 3/12) or severe AD (7.1%, 1/14), with a significant difference between mild and severe cases (*p* < 0.05). These findings suggest that severe AD is associated with higher colonization by SEA-producing CoNS and lower colonization by non-toxigenic strains (Figure 2).

### 3.3. Antibiotic Susceptibility of CoNS vs. CoPS

**Figure 3:**
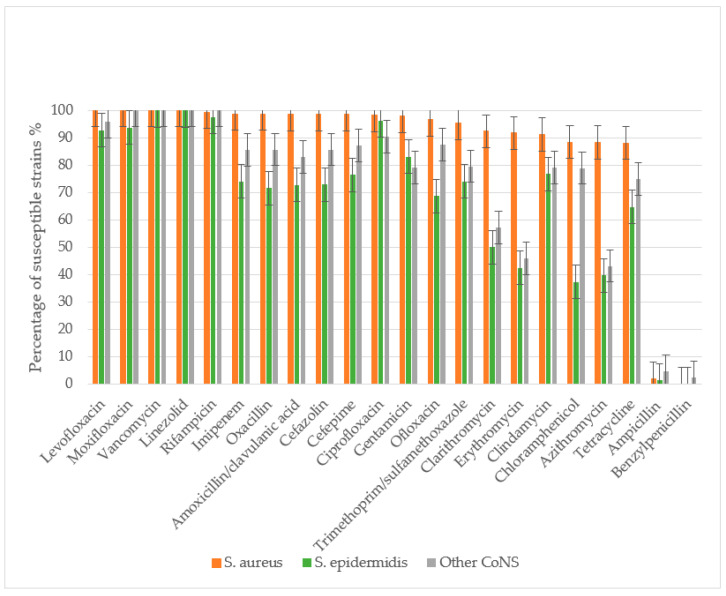
*Staphylococcus* spp. antibiotic susceptibility in descending order for *S. aureus*. *S. aureus* demonstrated absolute sensitivity to vancomycin, linezolid, and later-generation fluoroquinolones (levofloxacin and moxifloxacin), with a low MRSA prevalence of 1.27% as determined by oxacillin resistance. *S. epidermidis* showed significantly more resistant properties to most antibiotics, including β-lactams, macrolides, cephalosporins, tetracyclines, aminoglycosides, lincosamides, and fluoroquinolones (*p* < 0.05). The incidence of MRSE was 28.4%.

The antibacterial susceptibility of 160 *S. aureus* strains, 87 *S. epidermidis* strains, and 53 other CoNS strains (excluding *S. epidermidis*) was evaluated. The study assessed sensitivity to the following antibacterial agents: β-lactam antibiotics including penicillins (benzylpenicillin, ampicillin, amoxicillin-clavulanate, oxacillin), cephalosporins (cefazolin, cefepime), and carbapenems (imipenem); macrolides (azithromycin, clarithromycin, erythromycin); fluoroquinolones (ciprofloxacin, ofloxacin, levofloxacin, moxifloxacin); glycopeptides (vancomycin); lincosamides (clindamycin); rifamycins (rifampicin); tetracyclines (tetracycline); amphenicols (chloramphenicol); sulfonamides with trimethoprim (co-trimoxazole); aminoglycosides (gentamicin); and oxazolidinones (linezolid) (Figure 3).

All *S. aureus* and *S. epidermidis* strains demonstrated complete resistance to benzylpenicillin, with only minimal sensitivity retained to ampicillin. Resistance rates to ampicillin were 97.9% for *S. aureus*, 98.9% for *S. epidermidis*, and 95.9% for other CoNS. *S. aureus* maintained high sensitivity to amoxicillin/clavulanate, with only 1.38% of strains showing resistance. The prevalence of MRSA was low at 1.27%. In contrast, *S. epidermidis* and other CoNS exhibited significantly higher resistance to amoxicillin/clavulanate (27.2% and 17.03%, respectively), with methicillin-resistant strains reaching 28.4% (MRSE) and 14.2% (*p* < 0.01 compared to *S. aureus*).

*S. aureus* showed high susceptibility to cephalosporins, with resistance to both cefazolin and cefepime observed in only 1.27% of strains. Significantly higher resistance rates were noted in CoNS: 27.0% (*S. epidermidis*) and 14.3% (other CoNS) to cefazolin, and 23.5% and 12.8% to cefepime, respectively.

CoNS demonstrated substantially greater resistance to macrolides than *S. aureus* (*p* < 0.01). Resistance rates among *S. epidermidis* ranged from 50% (clarithromycin) to 60.3% (azithromycin), while other CoNS showed 42.8–56.8% resistance across macrolides. In contrast, *S. aureus* resistance remained low (8.16–11.62%), though still higher than its resistance to β-lactam/β-lactamase inhibitor combinations and cephalosporins.

For fluoroquinolones, *S. aureus* maintained high susceptibility, with only 1.86–3% resistance to second-generation agents (ofloxacin, ciprofloxacin) and complete sensitivity to third- and fourth-generation fluoroquinolones (levofloxacin, moxifloxacin). CoNS showed significantly higher resistance, particularly *S. epidermidis* (31.25% to ofloxacin). Resistance to newer fluoroquinolones was lower (3.71–7.32% in *S. epidermidis*, 4.17–12.5% in other CoNS), with all CoNS remaining susceptible to moxifloxacin.

All staphylococcal strains retained sensitivity to reserve antibiotics vancomycin and linezolid. Resistance to rifampicin and imipenem was rare in *S. aureus* (0.63–1.26%) but more prevalent in CoNS (25.97% of S. epidermidis and 14.59% of other CoNS to imipenem). While 2.46% of *S. epidermidis* showed rifampicin resistance, other CoNS maintained complete susceptibility.

CoNS exhibited significantly greater resistance to most antibiotic classes (*p* < 0.05), including β-lactams, macrolides, cephalosporins, tetracyclines, aminoglycosides, lincosamides, and fluoroquinolones. No association was found between disease severity (mild, moderate, severe) and antibiotic resistance patterns for any staphylococcal species when tested against all studied antimicrobial agents.

## 4. Discussion

Our study revealed distinct patterns of staphylococcal colonization in AD patients. *S. aureus* colonization was significantly more prevalent in severe AD cases, while CoNS strains, particularly *S. epidermidis*, dominated in mild AD. Although CoNS strains were less frequently detected on the skin of severe AD patients, they retained the ability to produce toxins. For certain toxins (SEA), the detection frequency in culture filtrates of strains isolated from severe AD patients was higher than in strains from mild AD cases. This suggests that despite the lower isolation rate of CoNS in severe AD, they may play a significant role in sustaining inflammation through toxin production.

CoNS strains isolated from the skin of children with AD, regardless of disease severity, demonstrated the ability to produce enterotoxins in vitro. Most CoNS strains produced multiple enterotoxins simultaneously. The number of CoNS strains with no detectable toxins in their culture filtrates was slightly higher compared to *S. aureus*, though this difference was not statistically significant. SEB and TSST-1 were the most frequently detected toxins in culture filtrates of both *S. aureus* and *S. epidermidis*, occurring with equal frequency across strains. SEB was detected in 1:10 dilutions of bacterial filtrates from a larger proportion of *S. aureus* strains. The maximum dilution at which TSST-1 was detectable was 1:10 for both *S. aureus* and CoNS, with equal detection frequency in *S. aureus* and *S. epidermidis*, but less frequently in other CoNS species.

SEA was detected less frequently than SEB and TSST-1. In undiluted culture filtrates, it occurred equally in *S. aureus* and CoNS. At 1:10 dilution, it was less frequent in CoNS except *S. epidermidis*. At 1:50 dilution, SEA was detectable only in *S. aureus* filtrates and a single *S. simulans* strain. This indicates that in vitro, CoNS strains from AD patients generally produce less enterotoxin A than *S. aureus*, though certain strains produce quantities comparable to *S. aureus*. The proportion of CoNS strains with detectable SEA was statistically higher in the severe AD group. Conversely, the number of CoNS strains with no detectable toxins was significantly lower in severe AD.

SEC was the least frequently detected toxin in undiluted filtrates. It occurred significantly more often in *S. aureus* filtrates than in CoNS.

Our data align with previous studies confirming that enterotoxigenic potential of CoNS may influence the disease course. In a 2017 study by Hon, K. L et al. in a pediatric population, it was demonstrated that serum anti-SEB IgE levels were positively associated with *S. aureus* and/or *S. epidermidis* skin isolation and objective SCORAD, as well as with clinical signs and quality of life [20]. Toxin production is not the only mechanism negatively influencing AD course. While traditionally viewed as a natural antagonist of *S. aureus*, certain *S. epidermidis* strains appear less capable of inhibiting *S. aureus* virulence in AD patients [28]. Genomic analyses reveal strain-specific differences in histopathological potential, antibiotic resistance profiles (including methicillin resistance), and immunomodulatory capacity [29].

Phylogenetic studies show striking differences between staphylococcal species in AD: while *S. aureus* exhibits clonal expansion, *S. epidermidis* demonstrates remarkable phylogenetic diversity across all disease stages. Mild AD cases predominantly harbor clades A29 and A30 strains, contrasting with the A20 dominance in healthy adults [30]. Notably, AD skin often lacks protective CoNS strains (*S. epidermidis* and *S. hominis*) that produce anti-*S. aureus* antimicrobial peptides [29]. Instead, AD-derived *S. epidermidis* strains exhibit pathogenic potential through multiple mechanisms. *S. epidermidis* strains isolated from lesional AD skin exhibit distinct pathogenic properties that significantly alter epidermal structure and function. Unlike commensal strains from healthy skin, AD-associated preferentially activates STAT6 while suppressing the protective AhR/OVOL1 pathway, accompanied by significantly reduced indole production [31]. These changes lead to marked downregulation of key differentiation markers, including filaggrin and desmoglein-1, compromising epidermal barrier function. AD-derived *S. epidermidis* strains exhibit other proinflammatory properties. Production of cytotoxic phenol-soluble modulins that show a strong positive correlation with disease severity (r = 0.78, *p* < 0.001) [32]. Secretion of the cysteine protease EcpA, which effectively degrades both desmoglein-1 (by 62 ± 8%) and the antimicrobial peptide LL-37 in vitro, thereby impairing physical barrier function and promoting skin inflammation [33]. Generation of extracellular serine protease that triggers IL-13 activation (3.5-fold increase) and drives a Th2-polarized immune response, characteristic of AD pathogenesis [34]. These findings collectively demonstrate that specific *S. epidermidis* strains possess multiple virulence factors capable of exacerbating AD through both direct barrier disruption and immune modulation. Not all *S. epidermidis* strains can inhibit *S. aureus* biofilm formation. In some patients, these species coexist, forming mixed-species biofilms that enhance their survival and pathogenicity [35].

Our study revealed a high prevalence of antibiotic resistance among CoNS. A 2017 species-level analysis of AD flares demonstrated that MRSA was more common in severe cases, whereas MRSE predominated in milder forms [28]. Supporting this, a 2023 genome-wide association (GWA) study reported MRSA in 13.79% and MRSE in 39% of cases [16]. These rates are significantly higher than we observed in our study. *S. epidermidis* can function as a reservoir for methicillin resistance that can be passed on to other species, including *S. aureus*; through the action of the recombinase genes in SCCmec, the cassette can be excised from the genome and transferred between isolates and between species. Plasmid-mediated resistance to tetracycline, streptomycin, and erythromycin can also drive dissemination from *S. epidermidis* to other species [36]. Given the potential influence of CoNS on the inflammatory process in AD, particularly through toxin production, we cannot ignore its multidrug resistance when selecting therapy.

## 5. Conclusions

This study elucidates the complex dynamics of staphylococcal colonization, virulence, and antibiotic resistance in pediatric AD. *S. epidermidis* may play a more significant role in AD than previously thought. Critically, CoNS strains from severe AD demonstrate enhanced enterotoxigenic activity (notably SEA production). Our results emphasize the need for further research on staphylococcal dynamics in AD and the potential for targeted microbial interventions. Specifically, alongside conventional anti-inflammatory therapy, future treatments should consider agents that modulate bacterial colonization—suppressing pathogenic strains while restoring beneficial flora during both active disease and remission. This study advances our understanding of AD etiology and may guide novel therapeutic strategies.

This study has potential limitations. The culture-based approach used in this study cannot fully capture the heterogeneity of the skin commensal flora, as it primarily targets easily cultivable microorganisms (*Staphylococcus* spp.). This limits our ability to assess non-culturable or fastidious organisms within the community. DNA sequencing techniques are required for comprehensive identification of difficult-to-cultivate microorganisms. WgMLST of isolated *Staphylococcus* spp. genomes was not performed. Such analysis is essential to confirm the prevalence and genetic context of mobile elements encoding superantigens across staphylococcal species. Putative methicillin-resistant strains identified phenotypically were not validated via mecA gene detection. This study lacks a healthy control group for comparative analysis of microbial composition and toxin prevalence. Future work will include healthy controls, prioritizing cohabiting relatives of AD patients to account for shared environmental exposures.

## Figures and Tables

**Table 1:** Demographics of AD patients and *Staphylococcus* spp. skin colonization.

Atopic Dermatitis Patients				
	Mild	Moderate	Severe	Total
Patients *n* (%)	68 (20.6%)	124 (37.7%)	137 (41.6%)	329
Male *n* (%)	43 (25.8%)	53 (31.9%)	70 (42.1%)	166
Female *n* (%)	25 (15.3%)	71 (43.5%)	67 (41.1%)	163
Age (yr)	2.9 ± 0.6	5.3 ± 0.9	5.5 ± 3.1	4.89 ± 0.9
SCORAD	15.1 ± 4.2	38.8 ± 4.7	71.1 ± 6.5	47.8 ± 5.1
Staphylococci strais	61 (20.4%)	109 (36.3%)	130 (43.3%)	300
*S. aureus*	22 (13.7%)	55 (34.3%)	83 (51.8%)	160 (53.3%)
CoNS total	39 (27.8%)	54 (38.5%)	47 (33.5%)	140 (46.6%)
*S. epidermidis*	24 (27.5%)	39 (44.8%)	24 (27.5%)	87
*S. haemolyticus*	4 (18.18%)	8 (36.36%)	10 (45.45%)	22
*S. hominis*	9 (60.0%)	4 (26.6%)	2 (13.3%)	15
*S. warneri*	1	1	3	5
*S. saprophyticus*	1			1
*S. capitis*			7	7
*S. simulans*		1		1
*S. cohnii*		1	1	2
No staphylococcal growth	20 (23.5%)	33 (38.8%)	32 (37.6%)	85

## Data Availability

The data presented in this study are available on request from the corresponding author due to privacy reasons.

## Abbreviations

The following abbreviations are used in this manuscript:

AD	Atopic dermatitis
CoNS	Coagulase-negative staphylococci
MRSA	Methicillin-resistant *Staphylococcus aureus*
MRSE	Methicillin-resistant *Staphylococcus epidermidis*
CoPS	Coagulase-positive staphylococci
SEA	Staphylococcal enterotoxin A
SEB	Staphylococcal enterotoxin B
SEC	Staphylococcal enterotoxin C
TSST-1	Toxic shock syndrome toxin 1
